# Regulation of Quality and Phenolic Metabolism in Apricot Fruit by Postharvest Salicylic Acid and Putrescine Applications

**DOI:** 10.1002/fsn3.72026

**Published:** 2026-06-14

**Authors:** Mustafa Sakaldaş, Fatih Şen, Muttalip Gundogdu, Erdal Aglar

**Affiliations:** ^1^ Department of Food Processing, Vocational School of Lapseki Çanakkale Onsekiz Mart University Çanakkale Türkiye; ^2^ Department of Horticulture, Faculty of Agriculture Ege University İzmir Türkiye; ^3^ Department of Park and Horiculture, Yalova Vocational School Yalova University Yalova Türkiye; ^4^ Department of Horticulture, Faculty of Agriculture Bolu Abant Izzet Baysal University Bolu Turkey; ^5^ Department of Landscape Architecture, Faculty of Arts, Design and Architecture Munzur University Tunceli Türkiye

**Keywords:** organic acids, phenolic compounds, respiration rate, salicylic, vitamin C

## Abstract

Apricots are a highly sensitive product that suffers rapid quality losses due to physiological and biochemical processes such as increased respiration, ethylene production, cell wall breakdown, and changes in phenolic compounds that occur in the post‐harvest period, and this significantly shortens their shelf life. This study evaluated the effects of salicylic acid (SA) and putrescine (Put) applications on post‐harvest quality characteristics, organic acid metabolism, and phenolic compound stability in apricot ( *Prunus armeniaca* L.) fruits. Fruits harvested at commercial maturity were treated with four different applications: control, SA (1 mM), Put (1 mM), and a combination of SA and Put. In this study, the physiological quality parameters such as weight loss, decay rate, soluble solids content (SSC), titratable acidity (TA), pH, and respiration rate were measured, and biochemical analyses of organic acids and individual phenolic compounds were performed. With increasing storage time, weight loss, decay rate, and pH values increased in all treatment groups, whereas significant decreases occurred in organic acids, ascorbic acid, and total phenolic compounds. However, it was determined that SA and Put applications significantly slowed down these negative changes. In particular, the SA + Put combination improved storage stability by providing the lowest weight loss and decay rate thanks to the preservation of cell membrane stability and the limitation of transpiration. Phenolic compound analyses showed that phenolic degradation is the natural process during storage, but SA and Put applications significantly slowed this process. In particular, it was determined that the combination application contributed to the preservation of antioxidant phenols by supporting phenylpropanoid metabolism, and that the highest level of phenolic stability was achieved in this group. PCA results revealed that quality degradation parameters were positively correlated with respiration rate and weight loss, whereas phenolic compounds, organic acids, and ascorbic acid showed strong positive correlations with quality preservation. Correlation analysis also supported these findings, confirming that antioxidant compounds play a decisive role in storage performance. In conclusion, it was determined that the combined application of SA and Put reduces oxidative stress, regulates metabolic activity, stabilizes phenolic metabolism, and significantly delays post‐harvest quality losses in apricot fruit. This approach was found to offer an effective, feasible, and sustainable post‐harvest strategy for extending shelf life and preserving nutritional quality components.

## Graphical abstract

This study evaluated the effects of salicylic acid and putrescine (1 mM) treatments on the quality and biochemical properties of apricot fruit during 30 days of cold storage. Although weight loss and decay increased during storage, applications significantly reduced these negative effects. Reductions in organic acids, vitamin C, and phenolic compounds were also significantly limited. PCA and correlation analyses showed strong relationships between quality parameters and biochemical components. In conclusion, Put and SA applications are an effective natural method for preserving post‐harvest quality in apricot fruit.

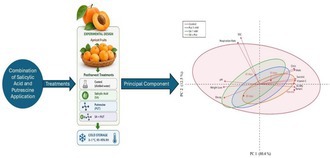

## 1 Introduction

Today, the increasing global population, climate change, and sustainability issues in agricultural production have made food and nutrition security a global priority. In this context, improving pre‐harvest and post‐harvest practices, especially in fruits and vegetables, is of great importance in reducing product losses and extending shelf life. Many studies have shown that pre‐ and post‐harvest applications of salicylic acid and its derivatives are effective in preserving quality and extending storage time in fruits and vegetables (Chen et al. 2023). In addition, increasing the nutritional value of plant products through biofortification and omics approaches is among the important strategies in ensuring nutrition security (Younas et al. 2023; Umar et al. 2023). Sustainable agricultural practices, pre‐treatment technologies, and innovative preservation methods play a critical role, especially in reducing post‐harvest losses (Joshi et al. 2024). Furthermore, the positive effects of nanotechnological applications on plant disease control, yield increase, and quality preservation have also attracted attention in recent years (Maqbool et al. 2025). New technologies and practices developed to preserve quality in the post‐harvest period have been successfully applied to various products such as grapes, apricots, carrots, and peppers, contributing to extended shelf life (Mushtaq et al. 2024; Moradinezhad and Jahani 2016; Saleem et al. 2026; Tariq et al. 2025). In this context, reducing post‐harvest quality losses and increasing product durability are of great importance both for preventing economic losses and ensuring food safety.

Fresh fruits constitute an important component of human nutrition due to their rich nutritional content and numerous bioactive compounds that contribute to human health. Among these fruits, apricots ( *Prunus armeniaca* L.) hold a significant place in terms of production and consumption worldwide thanks to their pleasant aroma, attractive color, and high nutritional value. Apricot fruits are particularly rich in phenolic compounds, carotenoids, organic acids, sugars, and vitamins, and these compounds contribute to the fruit's antioxidant capacity and potential health benefits (Bureau et al. 2009; Erdogan‐Orhan and Kartal 2011; Bartolini et al. 2015). Phenolic compounds such as chlorogenic acid, catechin, epicatechin, quercetin derivatives, and kaempferol play a significant role in determining both the nutritional quality and antioxidant activity of apricot fruits. These metabolites not only play a role in the defense mechanisms of plants but also contribute to the preservation of characteristics such as color, aroma, and taste that determine fruit quality (Manach et al. 2004; Scalbert et al. 2005; Williamson 2017; Watson and Preedy 2018). Despite their nutritional benefits and commercial importance, apricot fruits are quite delicate and susceptible to rapid quality loss during post‐harvest storage. As the climacteric fruit, apricots undergo rapid physiological and biochemical changes after harvest, such as increased respiration rate, elevated ethylene production, and accelerated metabolic activity. These processes result in rapid softening, discoloration, loss of organic acids, and degradation of bioactive compounds, significantly limiting the fruit's post‐harvest shelf life (Kim et al. 2004; Noppakoonwong et al. 2005; Leida et al. 2011). Furthermore, the high metabolic activity of apricot fruits during storage can lead to significant post‐harvest losses, posing a major problem for producers and the supply chain. Post‐harvest spoilage in fruits is largely related to oxidative stress caused by the accumulation of reactive oxygen species (ROS). The increased respiration and metabolic activity during storage increase ROS production in fruit tissues. Excessive accumulation of ROS can cause oxidative damage to cellular structures, such as lipid peroxidation in cell membranes, protein oxidation, and nucleic acid degradation (Blokhina et al. 2003; Gill and Tuteja 2010). This oxidative damage accelerates aging processes, leading to a decrease in quality characteristics such as texture, color, and nutritional value in the fruit tissue. Furthermore, ROS accumulation contributes to the breakdown of phenolic compounds and other antioxidants, resulting in a reduction in the fruit's antioxidant capacity. To mitigate these negative effects and extend the shelf life of fresh fruit, various post‐harvest technologies have been developed, including cold storage, controlled atmosphere storage, modified atmosphere packaging, edible coatings, and chemical applications. Among these methods, low‐temperature storage is one of the most commonly used to slow down metabolic processes and delay fruit aging. By reducing respiration rate and ethylene production, cold storage can significantly extend the storage life of apricots (Sharma et al. 1992). However, apricots are sensitive to low temperatures and can develop signs of cold damage during prolonged cold storage. These symptoms include problems such as internal browning, tissue deterioration, water loss, and surface collapse, which negatively affect the commercial value of the fruit (Stanley et al. 2013). Therefore, there is increasing interest in alternative strategies that can improve post‐harvest quality while reducing cold damage and oxidative stress. In recent years, there has been an increase in research on the use of natural compounds and plant‐derived regulators as alternatives to synthetic chemicals in post‐harvest preservation practices. Consumer concerns about chemical residues and environmental sustainability requirements have encouraged the development of safer and more sustainable technologies for preserving fruit quality. Among these approaches, the use of plant growth regulators and signaling molecules stands out as the promising strategy for regulating physiological processes in harvested fruits.

SA is the naturally occurring phenolic compound found in plants and is known as a signaling molecule that plays an important role in plant defense and stress responses. It plays a significant role in regulating many physiological processes in plants, such as growth, development, thermogenesis, and systemic acquired resistance (SAR) to pathogens (Hayat et al. 2010; Nazar et al. 2015). Furthermore, SA has been shown to regulate many metabolic pathways associated with fruit ripening and senescence processes. Numerous studies have revealed that post‐harvest SA applications delay fruit ripening, reduce respiration rate, and suppress ethylene biosynthesis (Asghari and Aghdam 2010; Ahmed et al. 2017). The positive effects of SA on fruit quality are largely due to its ability to regulate antioxidant metabolism and strengthen the plant defense system. SA applications have been reported to reduce ROS accumulation and oxidative damage by increasing the activities of antioxidant enzymes such as superoxide dismutase (SOD), catalase (CAT), and ascorbate peroxidase (APX) (Zhang et al. 2022). Thus, SA applications contribute to the preservation of cellular integrity and the delay of the aging process in post‐harvest fruits. Furthermore, it has been reported that SA delays fruit softening by suppressing the activity of enzymes involved in cell wall degradation such as polygalacturonase, cellulase, and pectin methylesterase (Shi et al. 2019). By suppressing these enzymes, fruit firmness and tissue stability can be maintained throughout storage. Another important effect of SA is its regulatory role on phenolic metabolism. It has been reported that SA applications can stimulate the phenylpropanoid metabolic pathway, increasing the synthesis of phenolic compounds and flavonoids in plant tissues. These compounds, as powerful antioxidants, protect cells against oxidative stress and enhance the nutritional value of fruits (Ehtesham Nia et al. 2022). Therefore, SA applications are widely researched both to improve post‐harvest fruit quality and to preserve the health benefits of fruits.

In addition to SA, polyamines are also noteworthy compounds that play an important role in the regulation of plant growth and stress tolerance. Polyamines are low molecular weight aliphatic amines and are widely found in plant cells, participating in various physiological processes such as cell division, differentiation, and senescence (Kusano et al. 2008; Gill and Tuteja 2010). The most common polyamines in plants are Put, spermidine, and spermine. These compounds play the role in regulating plant development and responses to environmental stresses. Among these polyamines, Put has been identified as an important regulator in fruit development, ripening, and post‐harvest physiology. Put enhances the stability of cell membranes by interacting with negatively charged molecules such as nucleic acids, proteins, and phospholipids, thus contributing to the preservation of the structural integrity of plant cells (Valero et al. 2002; Malik and Singh 2005). Post‐harvest Put applications have been reported to delay fruit ripening and extend storage time in many fruit species. For example, studies have shown that Put applications reduce weight loss, maintain fruit firmness, and decrease spoilage rates during storage (Hosseini et al. 2017; Hanif et al. 2020). These effects are largely due to putrescine's properties of reducing respiration rate, suppressing ethylene production, and maintaining cell membrane stability. Another important function of Put is the regulation of the antioxidant defense system. Post‐harvest Put applications have been reported to neutralize ROS by increasing the activity of antioxidant enzymes such as superoxide dismutase (SOD) and catalase (CAT), and to reduce oxidative damage in fruit tissues (Ba et al. 2025). Through this mechanism, Put applications contribute to delaying aging processes during storage by maintaining cellular balance. Put also plays the important role in regulating cell wall metabolism. The softening that occurs during fruit ripening is largely related to the breakdown of cell wall polysaccharides such as pectin, cellulose, and hemicellulose. Enzymes such as polygalacturonase, cellulase, and pectin methylesterase cause the breakdown of these structural components, leading to a decrease in fruit firmness (Brummell 2005). Various studies have shown that Put applications can maintain cell wall stability by suppressing the activity of these enzymes and can ensure that fruit firmness is maintained for a longer period during storage (Ba et al. 2025). In addition to its role in antioxidant metabolism and cell wall regulation, Put is also reported to have an effect on phenolic metabolism. Phenolic compounds are secondary metabolites that play the important role in antioxidant activity and defense mechanisms in plants. The maintaining high levels of phenolic compounds during storage is of great importance for preserving fruit quality and nutritional value. Studies have shown that Put applications can slow down the degradation of phenolic compounds, increasing their accumulation in fruit tissues (Kibar et al. 2021; Puente‐Moreno et al. 2026).

Although many studies have examined the effects of SA or polyamines on post‐harvest fruit quality, research on the combined application of these compounds is limited. Simultaneous application of different bioactive compounds may create synergistic effects in regulating physiological and biochemical processes in fruits. The combined treatments can enhance antioxidant capacity by increasing the activation of antioxidant defense systems, strengthening phenolic metabolism, and contributing to better preservation of fruit quality during storage.

Recent studies have shown that the combined use of different post‐harvest treatments can yield more effective results in controlling fruit senescence compared to individual treatments. For example, a combination of different plant regulators can simultaneously regulate respiratory metabolism, antioxidant defense systems, and phenolic biosynthesis pathways. Such holistic approaches offer a more comprehensive strategy for reducing post‐harvest losses and preserving fruit quality. Given the significant role of phenolic compounds in determining fruit quality and nutritional value, understanding the mechanisms regulating phenolic metabolism during post‐harvest storage is of great scientific and practical importance. In this context, the combined application of SA and Put is considered a promising approach for improving post‐harvest fruit quality by controlling oxidative stress and regulating phenolic metabolism. Therefore, the aim of this study is to investigate the effects of SA and Put applications on post‐harvest quality characteristics and phenolic metabolism in apricot fruit during storage. Specifically, the study aims to determine how these applications regulate physiological and biochemical processes associated with fruit senescence, antioxidant activity, phenolic compound stability, and overall fruit quality parameters.

## 2 Materials and Methods

### 2.1 Plant Material

In this study, the apricots of the “Kabaaşı” variety ( *P. armeniaca* L.), harvested at commercial maturity, were used as plant material. The fruits were obtained from trees in the same orchard with similar age and developmental characteristics. Harvesting was carried out in the morning hours, and care was taken to ensure that the fruits reached a homogeneous level of maturity. During harvesting, criteria such as fruit color development, size, surface smoothness, and the absence of any mechanical damage were considered. The harvested fruits were placed in plastic crates and transported to the laboratory as quickly as possible. The fruits brought to the laboratory were classified, and those showing mechanical damage, cracks, disease symptoms, or deformations were excluded from the experiment. The fruits to be used in the analyses were randomly distributed into experimental groups.

### 2.2 Treatment

The study consisted of four different treatment groups: control (distilled water), salicylic acid (SA, 1 mM), putrescine (Put, 1 mM), and a combination of SA and Put (SA 1 mM + Put 1 mM). The SA and Put solutions used in the experiment were prepared using analytical grade chemicals. Due to the limited solubility of the compound in water, the SA solution was first dissolved in a small amount of ethanol, then the volume was completed with distilled water to prepare a solution at the concentration of 1 mM. The Put solution was prepared at the same concentration by dissolving it directly in distilled water. In the combined application, SA and Put were prepared together in the same solution at a concentration of 1 mM. Harvested apricot fruits were randomly distributed into treatment groups, and the fruits in each treatment group were treated with the prepared solutions using the dip treatment method. The fruits were left in the respective solutions for 5 min and then left at room temperature until their surfaces dried to remove excess solution. Fruits in the control group were kept in distilled water only for the same period. The experiment was conducted in triplicate, with approximately 30 fruits used in each replicate. HPLC analyses were performed in triplicate. After the processing was completed, the fruits were placed in polyethylene crates and stored in a cold storage facility. The storage was carried out at a temperature of 0°C–1°C and a relative humidity of 90%–95%, with a total storage period of 30 days. To determine the physiological and biochemical changes occurring during storage, samples were taken at specific intervals. Analyses were conducted at four different storage times: on harvest day (Day 0), and on Days 10, 20, and 30. At each sampling time, randomly selected fruits from each treatment group were taken, and quality parameters and biochemical analyses were performed.

### 2.3 Weight Loss

To determine the physiological and biochemical changes occurring during storage, samples were taken at specific intervals. Analyses were conducted at four different storage times: on harvest day (Day 0), and on Days 10, 20, and 30. At each sampling time, randomly selected fruits from each treatment group were taken, and quality parameters and biochemical analyses were performed.

Weight loss:Initial weight−Storage weight/Initial weight×1000

### 2.4 Decay Rate

The rate of decay in fruits during storage was determined. At each sampling time, fruits were visually inspected, and those showing signs of fungal infection, soft rot, or any microbial spoilage were considered rotten. The decay rate was calculated by dividing the number of rotten fruits by the total number of fruits and expressed as a percentage (%).

### 2.5 SSC, TA, and pH

To perform SSC, TA, and pH analyses, the fruit juice extract was first prepared. For this purpose, the edible part of apricot fruits from each treatment group was separated from their pits, and the fruit pulp was broken down in a homogenizer to obtain the homogenous sample. The homogenized samples were filtered through cheesecloth or filter paper to obtain clear fruit juice. The obtained fruit juice extract was stored briefly under cold conditions for use during the analyses.

SSC in fruits was determined using fruit juice extract. For analysis, a few drops of the obtained fruit juice were placed on a digital refractometer (Atago, Japan) prism, and measurements were taken at room temperature. Before measurements, the refractometer was calibrated using distilled water. At least three measurements were taken for each sample, and the average of the obtained values was calculated. Results are expressed as percentages (%).

TA analysis was performed based on the titration of the fruit juice extract with the standard base solution. For this purpose, a specific volume of the filtered fruit juice sample was taken and a few drops of phenolphthalein indicator were added. The sample was then titrated with a 0.1 N NaOH solution, and the titration process was continued until the pH of the solution reached approximately 8.1. TA was calculated considering the amount of NaOH consumed, and the results were expressed as malic acid equivalent (%), the predominant organic acid in apricot fruit.

The pH value of the fruit juice extract was determined using a digital pH meter (Hanna Instruments, USA). Prior to measurements, the pH meter was calibrated using pH 4.0 and pH 7.0 buffer solutions. After calibration, measurements were taken by directly immersing the electrode in the fruit juice extract. At least three repeated measurements were taken for each sample, and the average of the obtained values was calculated to express the results in pH units.

### 2.6 Respiration Rate

The respiration rate of the fruits was determined by placing a certain quantity of fruit in airtight glass containers and measuring the amount of carbon dioxide (CO_2_) accumulated in the containers using a gas analyzer. The measured CO_2_ amount was calculated taking into account the fruit weight and incubation time, and the results were expressed in mL CO_2_ kg^−1^ h^−1^.

### 2.7 Organic Acids and Vitamin C

For organic acid and ascorbic acid analyses, fruit samples were homogenized in a homogenizer. A specific amount of the homogenized sample was taken and mixed with the appropriate extraction solution. The mixture was vortexed and then centrifuged at high speed. The resulting supernatant was passed through a 0.45 μm membrane filter to prepare it for HPLC analysis. The amounts of organic acids (malic acid, citric acid, tartaric acid, and succinic acid) and ascorbic acid were determined using high‐performance liquid chromatography (HPLC). Analyses were performed using a reversed‐phase C18 column. Acidified distilled water or a suitable buffer solution was used as the mobile phase. During the analysis, the flow rate was set to 1.0 mL min^−1^ and detection was performed in the wavelength range of 210–254 nm using a UV detector. Compound identification and quantitative analysis were performed using calibration curves created with analytical standards. Results are expressed as mg 100 g^−1^ fresh weight (Bevilacqua and Califano 1989; Kafkas et al. 2006; Cemeroglu 2010).

### 2.8 Individual Phenolic Compounds

For phenolic compound analyses, fruit samples were ground into a fine powder using liquid nitrogen. The ground samples were extracted with an extraction solution containing methanol, and the mixture was vortexed. The samples were then centrifuged to separate them from solid particles, and the resulting supernatants were passed through a membrane filter to prepare them for HPLC analysis. The analysis of phenolic compounds was performed using an HPLC‐DAD (diode array detector) system. The analyses identified phenolic compounds commonly found in apricot fruit. These included chlorogenic acid, gallic acid, syringic acid, catechin, rutin, ferulic acid, protocatechuic acid, p‐coumaric acid, caffeic acid, and o‐coumaric acid. Compound identification was performed by comparing retention times with standard substances. Quantitative analyses were carried out using calibration curves created with standard solutions. Results are expressed as mg 100 g^−1^ fresh weight (Rodríguez‐Delgado et al. 2001).

### 2.9 Statistical Analysis

The data obtained in this study were statistically evaluated to determine the effects of treatment and storage time factors. The experiment was conducted according to a two‐factor factorial design (treatments × storage time) and a randomized block design. Data obtained considering the treatment (Control, SA 1 mM, Put 1 mM, and SA + Put) and storage times (0th, 10th, 20th, and 30th day) were subjected to analysis of variance (ANOVA). The differences between means were determined using Duncan's multiple comparison test, and the differences were evaluated at a significance level of *p* ≤ 0.05. Results are expressed as mean ± standard error (SE). Furthermore, correlation analysis was performed to reveal the relationships between quality parameters and biochemical compounds, and principal component analysis (PCA) was applied to evaluate multivariate relationships between variables. All statistical analyses were performed using SPSS (Version 22.0; IBM Corp., USA) software.

## 3 Results and Discussion

### 3.1 Weight Loss

**FIGURE 1:**
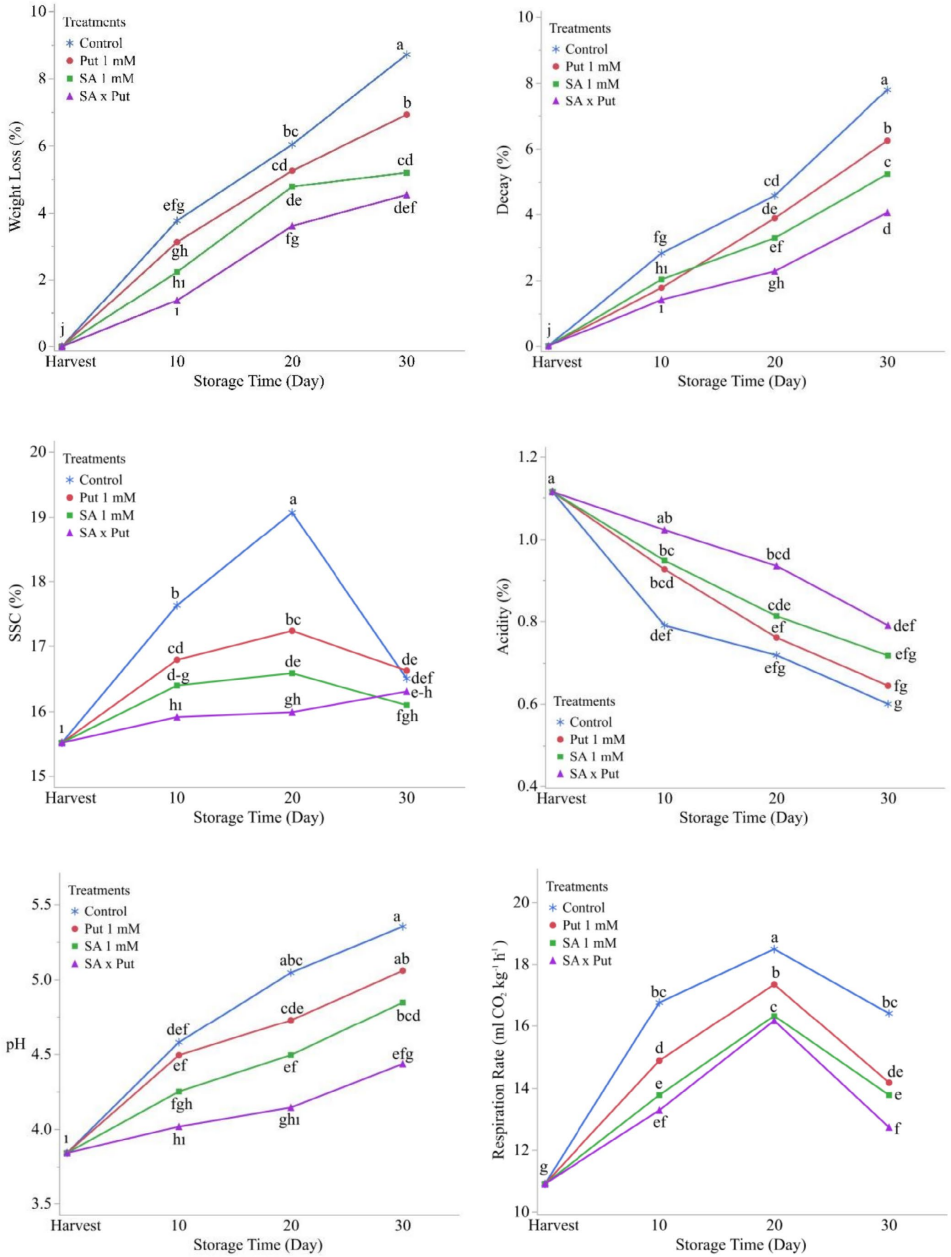
Changes in the quality characteristics of apricot fruits during storage depending on the treatments applied. Different letters on top of the storage periods indicate significant differences at *p* ≤ 0.05.

It was determined that there were significant increases in weight loss values in apricot fruits during storage (Table 1 and Figure 1). It was observed that the weight loss value gradually increased as the storage period progressed. When the average values were examined, it was determined that the weight loss reached 2.62% on the 10th day of storage, 4.91% on the 20th day, and 6.34% on the 30th day. The increase in weight loss was relatively limited during the initial storage period (0–10 days), it was observed to increase more significantly in the later stages of storage, particularly on Days 20 and 30. When comparing the treatments, the highest weight loss values were observed in the control group. At the end of storage, the weight loss in the control group reached 8.71%, whereas it was 5.19% in the SA treatment and 6.92% in the Put treatment. The lowest weight loss values in the study were obtained in the combination application of SA and Put. In this application, weight loss of 1.38%, 3.60%, and 4.53% was determined on the 10th, 20th, and 30th days of storage, respectively, and these values were found to be lower compared to other applications (Figure 1 and Table 1). These results show that weight loss in apricot fruits increases with the progress of storage time, but the applications have a significant effect on this loss. Weight loss in fruits during storage is primarily due to transpiration and respiration processes. Evaporation of water from the fruit tissue and carbon loss during respiration lead to a decrease in fruit mass during storage. This process is more pronounced in fruits with high water content, and significant water loss can occur during storage. Ongoing respiration and metabolic reactions in fruits after harvest can increase water loss, leading to quality losses in the fruit tissue (Alan et al. 2025; Kaygisiz et al. 2025). Therefore, increased weight loss during storage is an expected phenomenon in post‐harvest fruit physiology. The results obtained in this study show that SA and Put applications are effective in reducing weight loss. Put is one of the polyamines commonly found in plants and can limit water loss by increasing the stability of the cell membrane and protecting cellular structures. Furthermore, it has been reported that Put applications can reduce mass loss during storage by slowing down respiration and transpiration processes (Alan et al. 2025; Kucuker et al. 2025). It is stated that polyamines, thanks to their positively charged structures, interact with cell membranes and macromolecules, contributing to the maintenance of cellular stability and thus reducing physiological deterioration during storage (Kusano et al. 2008; Gill and Tuteja 2010). Similarly, SA is known to reduce quality losses by regulating post‐harvest fruit physiology. It is reported that SA delays fruit ripening by suppressing ethylene production and respiration rate, thus slowing down metabolic activities during storage (Asghari and Aghdam 2010; Ahmed et al. 2017). Furthermore, it is stated that SA protects the structural integrity of fruit tissue by reducing the activity of enzymes that cause cell wall breakdown, thereby limiting water loss (Shi et al. 2019). These mechanisms play an important role in contributing to the preservation of fruit quality during storage through SA applications. The studies on different fruit species in the literature also support these findings. Many studies have reported that Put applications can reduce weight loss in fruits during storage and contribute to the preservation of fruit quality (Wannabussapawich and Seraypheap 2018; Hanif et al. 2020). Similarly, studies on pomegranate fruit have reported that Put applications reduce physiological disorders occurring during storage and help preserve fruit quality parameters (Fawole et al. 2020). Furthermore, the study conducted on mulberry fruit determined that Put applications significantly reduced weight loss during storage by decreasing the respiration rate (Yavic et al. 2024). It has also been reported that SA applications can similarly reduce water loss and quality losses during storage in many fruit types (Kaygisiz et al. 2025). This study specifically determined that the combined application of SA and Put was more effective in reducing weight loss. It has been reported that the combined application of bioactive compounds can create synergistic effects in fruit tissue and activate antioxidant defense systems more strongly (Alan et al. 2025; Kaygisiz et al. 2025). This helps reduce oxidative stress and protect cellular structures, thus enabling better preservation of fruit quality during storage. Therefore, the findings suggest that the combination of SA and Put can be an effective application in limiting weight loss during apricot storage and preserving fruit quality.

**TABLE 1:** Changes in apricot fruit quality characteristics during storage due to salicylic acid and putrescine applications.

Storage time		Weight loss (%)	Decay (%)	SSC (%)	Acidity (%)	pH	Res. rate (mL CO_2_ kg^−1^ h^−1^)
Harvest		0.00 ± 0.00d	0.00 ± 0.00d	15.51 ± 0.30b	1.11 ± 0.09a	3.84 ± 0.14c	10.91 ± 0.48c
Day 10		2.62 ± 0.35c	2.01 ± 0.21c	16.68 ± 0.24ab	0.92 ± 0.04b	4.33 ± 0.09bc	14.67 ± 0.51b
Day 20		4.91 ± 0.37b	3.50 ± 0.34b	17.22 ± 0.44a	0.81 ± 0.03c	4.60 ± 0.13ab	17.08 ± 0.37a
Day 30		6.34 ± 0.64a	5.83 ± 0.53a	16.38 ± 0.09ab	0.69 ± 0.03d	4.92 ± 0.13a	14.27 ± 0.52b
Salicylic + putrescine and storage time interaction							
Harvest		0.00 ± 0.00j	0.00 ± 0.00j	15.51 ± 0.30ı	1.11 ± 0.09a	3.84 ± 0.14ı	10.91 ± 0.48g
Day 10	Control	3.76 ± 0.15efg	2.82 ± 0.16fg	17.63 ± 0.16b	0.79 ± 0.07def	4.58 ± 0.20def	16.76 ± 0.22bc
	SA 1 mM	2.23 ± 0.16hı	2.03 ± 0.18hı	16.39 ± 0.12d‐g	0.95 ± 0.02bc	4.25 ± 0.08fgh	13.77 ± 0.27e
	Put 1 mM	3.12 ± 0.28gh	1.77 ± 0.20hı	16.79 ± 0.18cd	0.93 ± 0.06bcd	4.49 ± 0.07ef	14.88 ± 0.21d
	SA + Put	1.38 ± 0.13ı	1.41 ± 0.10ı	15.91 ± 0.11hı	1.02 ± 0.04ab	4.01 ± 0.09hı	13.28 ± 0.28ef
Day 20	Control	6.03 ± 0.39bc	4.57 ± 0.43cd	19.07 ± 0.13a	0.72 ± 0.03efg	5.04 ± 0.11abc	18.49 ± 0.48a
	SA 1 mM	4.77 ± 0.67de	3.28 ± 0.27ef	16.58 ± 0.10de	0.81 ± 0.03cde	4.49 ± 0.10ef	16.32 ± 0.23c
	Put 1 mM	5.25 ± 0.35cd	3.89 ± 0.18de	17.24 ± 0.11bc	0.76 ± 0.05ef	4.72 ± 0.11cde	17.35 ± 0.31b
	SA + Put	3.60 ± 0.36fg	2.28 ± 0.27gh	15.98 ± 0.10gh	0.93 ± 0.03bcd	4.14 ± 0.09ghı	16.17 ± 0.16c
Day 30	Control	8.71 ± 0.71a	7.79 ± 0.26a	16.50 ± 0.16def	0.60 ± 0.03g	5.35 ± 0.09a	16.41 ± 0.47bc
	SA 1 mM	5.19 ± 0.23cd	5.22 ± 0.19c	16.09 ± 0.11fgh	0.72 ± 0.02efg	4.85 ± 0.06bcd	13.77 ± 0.21e
	Put 1 mM	6.92 ± 0.42b	6.24 ± 0.26b	16.62 ± 0.08de	0.64 ± 0.04fg	5.06 ± 0.10ab	14.17 ± 0.17de
	SA + Put	4.53 ± 0.18def	4.06 ± 0.17d	16.30 ± 0.16e‐h	0.79 ± 0.07def	4.43 ± 0.11efg	12.73 ± 0.29f
ANOVA							
*F* (storage time)		18.81***	25.92***	2.85^ns^	13.52***	7.5**	14.04***
*F* (SA × Put × storage time)		39.58***	87.04***	36.82***	9.34***	16.52***	46.59***

*Note:* Different letters in the same column indicates statistical differences at *p* ≤ 0.05.

Abbreviation: ns, not significant.

**p ≤ 0.01 and ***p ≤ 0.001, respectively.

### 3.2 Decay Rate

The significant increase in the rate of decay in apricot fruits was observed during storage. The rate of decay gradually increased as the storage period progressed. On the 10th day of storage, the decay rate reached 2.01%, rose to 3.50% on the 20th day, and reached 5.83% at the end of storage (30th day). This indicates that spoilage and microbial decay in apricot fruits progressively increase with longer storage periods. When the treatments were compared, it was determined that the highest decay rate occurred in the control group. At the end of storage, the decay rate in the control group was measured as 7.79%, which was higher than that of the other treatments. In contrast, SA and Put treatments were found to be effective in reducing the decay rate. However, the lowest decay rates were obtained in the combination application of SA and Put. In this application, decay rates of 1.41%, 2.28%, and 4.06% were determined on the 10th, 20th, and 30th days of storage, respectively, and these values were found to be lower than all other applications (Figure 1 and Table 1). These results show that SA and Put applications can significantly limit the development of decay in apricot fruits during storage. Fruit decay during storage is mostly related to physiological and biochemical changes occurring in the fruit tissue. Ongoing respiration, breakdown of cell wall components, and increased oxidative stress in the post‐harvest period can cause structural damage to the fruit tissue. During this process, increased permeability of cell membranes and leakage of cell contents create a suitable environment for the growth of microorganisms, accelerating the decay process. Furthermore, oxidative stress occurring in fruit tissue leads to the accumulation of reactive oxygen species, which can damage cellular structures and reduce resistance to pathogen development (Blokhina et al. 2003; Gill and Tuteja 2010). Therefore, an increase in the rate of spoilage in fruits is expected as the storage period lengthens. The study determined that SA and Put applications limited the development of decay. SA is a naturally occurring signaling molecule in plants and plays an important role in regulating plant defense mechanisms. When applied post‐harvest, SA is reported to contribute to reducing oxidative stress by increasing antioxidant enzyme activities in fruit tissues, thus helping to protect the cellular structure (Asghari and Aghdam 2010). It has also been stated that SA delays fruit ripening by reducing ethylene production and respiration rate, thus limiting quality losses during storage (Ahmed et al. 2017). In addition, it has been reported that SA exhibits antifungal properties that can suppress the growth of some pathogens and can reduce disease development by activating systemic acquired resistance mechanisms in plants (Amborabe et al. 2002). Put is one of the polyamines commonly found in plants and is the compound that plays an important role in maintaining cellular stability. It has been reported that Put applications increase the stability of the cell structure by interacting with cell membranes and cell wall components, thus contributing to making the fruit tissue more resistant during storage (Kusano et al. 2008; Gill and Tuteja 2010). It is also stated that Put applications delay fruit ripening by reducing respiration rate, thereby contributing to the reduction of physiological deterioration during storage (Alan et al. 2025). Furthermore, it is reported that polyamines can strengthen the antioxidant defense system, reducing oxidative damage caused by reactive oxygen species and thus helping to maintain structural integrity in fruit tissue (Ba et al. 2025). Previous studies have reported that Put applications can reduce the rate of fruit decay in the post‐harvest period and contribute to the preservation of fruit quality (Hanif et al. 2020). Similarly, studies on pomegranate fruit show that Put applications reduce physiological disorders occurring during storage and help preserve fruit quality parameters (Fawole et al. 2020). SA applications have also been reported to limit disease development in different fruit types, thereby reducing quality losses during storage. For example, studies on strawberries have shown that SA applications reduce ethylene production, lowering the rate of fungal decay and improving the fruit quality index (Babalar et al. 2007).

### 3.3 SSC, TA, and pH

It was determined that SSC values in apricot fruits changed over time during storage. The SSC value, measured at 15.51% at harvest, increased to 16.68% and 17.22% on the 10th and 20th days of storage, respectively, but decreased to 16.38% on the 30th day. Among the treatments, the highest SSC value was determined in the control group (19.07%) on the 20th day, and it was observed that the increase in SSC occurred more rapidly in the control fruits. Although the increase was more limited in the Put application, the SA application made the SSC change more balanced. The most stable change was observed in the SA + Put combination; SSC values remained within a narrow range throughout storage, determined as 15.91%, 15.98%, and 16.30%, respectively (Figure 1 and Table 1). The increase observed in SSC during the initial storage period can be explained by the accumulation of soluble sugars as a result of the hydrolysis of starch and complex carbohydrates during the maturation process. In the later stages of storage, the continuation of respiratory metabolism and the use of sugars in energy production cause a decrease or stabilization in SSC values. The higher SSC increase in the control group indicates a faster progression of respiration rate and metabolic activity. In contrast, Put application is known to delay maturation by reducing respiration rate and slowing down carbohydrate metabolism (Alan et al. 2025). Salicylic acid's regulatory effect on ethylene production and its activation of the antioxidant defense system also contribute to more controlled metabolic reactions. The more stable SSC change observed in combined application can be attributed to the synergistic effect of bioregulators in suppressing respiration and maturation processes. Similarly, previous studies have reported that the combined application of bioregulators reduces quality losses by maintaining metabolic balance (Kaygisiz et al. 2025).

It was determined that the TA values in apricot fruits decreased steadily during the storage period. The acidity value, which was 1.11% at harvest, decreased to 0.92%, 0.81%, and 0.69% on the 10th, 20th, and 30th days of storage, respectively. The lowest TA value was measured in the control group (0.60%) at the end of storage, and it was determined that Put and SA applications limited the acidity loss. The highest TA values were obtained with the SA + Put application and were maintained at 0.79% at the end of the 30th day (Figure 1 and Table 1). Faster acid loss in the control group can be considered an indicator of higher metabolic activity and accelerated aging. Putrescine is known to slow metabolic reactions and reduce respiration rate by increasing cell membrane stability (Kucuker et al. 2025). The maintaining acidity is crucial for preserving the sugar/acid balance and is a fundamental quality criterion that directly affects the perception of aroma, flavor, and freshness. Therefore, it can be said that the SA + Put application offers an advantage in terms of preserving sensory quality. SA has been reported to contribute to the preservation of biochemical compounds by suppressing ethylene synthesis and strengthening the antioxidant system. Therefore, it is thought that organic acids are consumed more slowly in fruits treated with SA. The preservation of TA values at the highest level in the combination application is consistent with the findings that the combined use of bioactive compounds reduces oxidative stress and limits metabolic consumption (Kaygisiz et al. 2025).

It was determined that pH values continuously increased during storage. The pH value, which was 3.84 at harvest, increased to 4.33, 4.60, and 4.92 on the 10th, 20th, and 30th days, respectively. The highest pH value was obtained in the control group (5.35) at the end of storage, whereas Put and SA applications were found to limit the pH increase. The lowest pH values were determined in the SA + Put application and remained at 4.43 on the 30th day (Figure 1 and Table 1). The increase in pH is associated with a decrease in the acidity of the fruit juice due to the breakdown of organic acids during respiration. Therefore, an inverse relationship between pH and TA is expected. The rapid pH increase in the control group indicates that organic acids are metabolized more quickly. Although putrescine slows down metabolic processes by increasing cellular stability and reducing oxidative damage (Alan et al. 2025), SA is reported to limit biochemical changes by strengthening antioxidant defenses (Kaygisiz et al. 2025). The fact that the pH increase remains at the lowest level in the SA + Put combination is associated with better preservation of organic acids. Previous studies also support the fact that the combined application of bioactive compounds maintains chemical balance in fruit tissue and slows down the aging process (Alan et al. 2025; Kaygisiz et al. 2025).

### 3.4 Respiration Rate

It was determined that the respiration rate of apricot fruits showed significant time‐dependent changes during the storage period. The respiration rate, measured as 10.91 mL CO_2_ kg^−1^ h^−1^ at harvest, increased in the early stages of storage, reaching 14.67 mL CO_2_ kg^−1^ h^−1^ on the 10th day and 17.08 mL CO_2_ kg^−1^ h^−1^ on the 20th day, and decreased to 14.27 mL CO_2_ kg^−1^ h^−1^ towards the end of storage. This indicates that respiratory activity initially increases and then decreases during the storage process, exhibiting a typical respiration pattern characteristic of climacteric fruits. When the treatments were compared, the highest respiration rates were determined in the control group, reaching a maximum particularly in the middle of storage (Day 20) with a value of 18.49 mL CO_2_ kg^−1^ h^−1^. It was observed that SA and Put applications limited the increase in respiration rate, with maximum values occurring at lower levels. The lowest respiration rates were determined in the SA + Put combination, and values of 13.28, 16.17, and 12.73 mL CO_2_ kg^−1^ h^−1^ were obtained on Days 10, 20, and 30 of storage, respectively (Figure 1 and Table 1). Since apricots are the climacteric fruit, the increase in respiration rate in the post‐harvest period is a natural part of the ripening process. During this process, the acceleration of metabolic reactions increases the breakdown of carbohydrates, and energy production rises, resulting in a temporary increase in respiration rate. In the later stages of storage, however, respiration activity decreases due to the reduction of metabolic substrates and tissue senescence (Kim et al. 2004; Noppakoonwong et al. 2005). SA is reported to slow down the maturation process by suppressing ethylene production and respiratory metabolism, and also contributes to reducing oxidative stress by increasing antioxidant enzyme activities (Asghari and Aghdam 2010; Ahmed et al. 2017). Put, as a polyamine, interacts with cell membranes and macromolecules, increasing cellular stability, reducing the accumulation of reactive oxygen species, and contributing to the slowing of metabolic activities (Kusano et al. 2008; Ba et al. 2025). The lower respiration rates observed with the combined use of SA and PUT applications can be explained by the fact that these two bioregulators act through different but complementary physiological mechanisms. Strengthening the antioxidant defense system, suppressing ethylene synthesis, and protecting cellular structure may have allowed for more controlled metabolic processes. Previous studies have shown that an increase in respiration rate during storage of climacteric fruits is one of the main factors accelerating quality losses (Noppakoonwong et al. 2005). It has been reported that SA applications regulate respiration rate, delay ripening, and contribute to the preservation of quality parameters during storage (Ahmed et al. 2017; Fan et al. 2021). Similarly, it has been reported that Put applications improve storage performance by reducing respiration rate in different fruit species. In the study conducted on mulberry fruit, it was determined that Put applications reduced respiration rate and limited quality losses (Yavic et al. 2024). In addition, the fact that polyamines reduce oxidative damage by activating antioxidant enzyme systems is considered one of the main reasons for metabolic slowdown (Ba et al. 2025).

### 3.5 Organic Acids and Vitamin C

The significant changes occurred in the organic acid composition and vitamin C content of apricot fruits during storage. The amounts of malic acid, citric acid, succinic acid, tartaric acid, and ascorbic acid were found to decrease regularly with increasing storage time. Statistical analyses showed that storage time, as well as the application × storage time interaction, had significant effects on all parameters (*p* ≤ 0.001). High levels of organic acids and vitamin C, observed during harvest, gradually decreased as storage progressed, with significant losses occurring particularly during the final storage period (Table 2). This trend is related to the apricot fruit maintaining its metabolic activity after harvest and can be explained by the consumption of organic acids in energy metabolism. The use of major organic acids such as malic and citric acid as substrates in the tricarboxylic acid cycle leads to a decrease in these compounds during storage. Similarly, the role of ascorbic acid in the defense mechanism against oxidative reactions leads to a decrease in vitamin C content as storage progresses (Kiralan and Gundogdu 2021). The fact that the fastest biochemical loss occurred in the control group among the treatments can be attributed to the higher respiration rate in fruits that did not receive any metabolic regulators. The faster depletion of organic acids and the more intense oxidative degradation of ascorbic acid in control fruits indicate that post‐harvest aging processes are accelerated. In contrast, SA application significantly supported the preservation of organic acids and limited vitamin C losses. SA is known to reduce respiration rate by suppressing ethylene biosynthesis and to decrease oxidative stress levels by activating antioxidant enzyme systems (Asghari and Aghdam 2010; Ahmed et al. 2017; Fan et al. 2021). Put application similarly contributed to maintaining biochemical stability. Polyamines are reported to maintain membrane integrity by interacting with cell membrane phospholipids and limiting the formation of reactive oxygen species. This leads to a slowing of metabolic reactions and a reduction in organic acid consumption (Gill and Tuteja 2010; Ba et al. 2025). However, it was observed that Put application alone did not provide as strong protection as SA in some parameters. The most remarkable result of the study was obtained in the combination of SA and Put application. In the SA + Put application, all organic acids and vitamin C content were preserved at higher levels during storage compared to other applications (Table 2). This indicates that the two bioregulators have a synergistic effect. The combined effect of SA activating the antioxidant system and Put enhancing cellular stability may have contributed to both slowing respiratory metabolism and limiting oxidative degradation. Previous studies have reported that bioregulator applications support the preservation of nutritional compounds by reducing post‐harvest quality losses. In particular, it has been shown in different fruit types that SA applications increase the stability of ascorbic acid and organic acids (Fan et al. 2021), whereas Put applications delay the degradation of phenolics and antioxidant compounds (Kibar et al. 2021; Tas et al. 2024). Strengthening the antioxidant defense system by polyamines is considered one of the main mechanisms of this effect (Ba et al. 2025).

**TABLE 2:** Modification in the organic acid and vitamin C content of apricot fruit during storage due to salicylic acid and putrescine treatments (mg 100 g^−1^).

Storage time		Malic	Citric	Succinic	Tartaric	Vitamin C
Harvest		943.10 ± 8.66a	735.85 ± 17.85a	20.34 ± 0.66a	29.90 ± 0.90a	48.53 ± 0.53a
Day 10		840.48 ± 7.19b	650.66 ± 8.95b	16.86 ± 0.57b	26.54 ± 0.81a	38.78 ± 0.71b
Day 20		775.23 ± 13.34c	620.77 ± 7.60c	13.83 ± 0.52c	21.63 ± 1.40b	34.19 ± 0.61c
Day 30		676.48 ± 11.48d	544.23 ± 12.22d	11.36 ± 0.62d	19.08 ± 1.33b	30.64 ± 0.74d
Salicylic + putrescine and storage time interaction						
Harvest		943.10 ± 8.66a	735.85 ± 17.85a	20.34 ± 0.66a	29.90 ± 0.90a	48.53 ± 0.53a
Day 10	Control	816.49 ± 5.21d	621.24 ± 7.46cd	14.93 ± 0.41cd	23.59 ± 0.57ef	36.08 ± 0.57d
	SA 1 mM	841.20 ± 3.00c	668.26 ± 11.81b	17.63 ± 0.25b	27.36 ± 0.13bc	39.91 ± 0.10b
	Put 1 mM	835.90 ± 3.87c	637.74 ± 5.76c	16.13 ± 0.40c	25.96 ± 0.55cd	38.22 ± 0.40c
	SA + Put	868.32 ± 5.02b	675.39 ± 7.69b	18.73 ± 0.52b	29.25 ± 0.68ab	40.93 ± 0.31b
Day 20	Control	720.95 ± 5.50g	589.84 ± 8.97e	12.32 ± 0.44e	15.61 ± 0.76h	31.75 ± 0.26g
	SA 1 mM	791.73 ± 3.04e	635.94 ± 3.24c	14.45 ± 0.51d	24.22 ± 0.61de	34.71 ± 0.25e
	Put 1 mM	772.40 ± 6.73f	619.58 ± 4.02cd	12.89 ± 0.31e	21.85 ± 0.34f	34.16 ± 0.20ef
	SA + Put	815.83 ± 4.99d	637.74 ± 5.76c	15.65 ± 0.52cd	24.84 ± 0.79de	36.12 ± 0.52d
Day 30	Control	632.78 ± 9.12j	513.93 ± 12.96g	9.10 ± 0.31g	14.48 ± 1.02h	28.33 ± 0.72h
	SA 1 mM	690.29 ± 5.84h	549.20 ± 6.48f	12.67 ± 0.31e	19.47 ± 0.39g	31.46 ± 0.17g
	Put 1 mM	670.02 ± 4.67ı	521.85 ± 6.48fg	10.61 ± 0.49f	18.20 ± 0.10g	29.47 ± 0.28h
	SA + Put	712.82 ± 9.51g	591.95 ± 14.34de	13.05 ± 0.40e	24.17 ± 0.76de	33.31 ± 0.39f
ANOVA						
*F* (storage time)		60.48***	34.99***	25.62***	9.88***	57.55***
*F* (SA × Put × storage time)		207.19***	42.3***	52.91***	57.35***	182.02***

*Note:* Different letters in the same column indicates statistical differences at *p* ≤ 0.05.

*p ≤ 0.05, **p ≤ 0.01, and ***p ≤ 0.001, respectively.

### 3.6 Individual Phenolic Compounds

It was determined that the vast majority of individual phenolic compounds identified in apricot fruits during the storage process showed a time‐dependent decrease. Statistical evaluations revealed that both storage time and the application × storage time interaction had a significant effect on all phenolic compounds examined (*p* ≤ 0.05). Overall, the findings show that the decrease in phenolic compounds was more pronounced in the control group, whereas SA and Put applications limited this decrease. Chlorogenic acid was one of the most abundant phenolic compounds in apricot fruit and showed a continuous decrease throughout storage. The value, which was 187.36 mg 100 g^−1^ at harvest, decreased to 130.74 mg 100 g^−1^ at the end of storage, whereas this decrease was more pronounced in the control group, falling to 120.71 mg 100 g^−1^. SA and Put applications contributed to the preservation of chlorogenic acid, with the highest values obtained in the SA + Put combination. Similarly, gallic acid, syringic acid, and catechin contents gradually decreased as storage progressed; losses were found to occur more rapidly, particularly in the control group. In contrast, higher levels of these compounds were observed in fruits treated with SA and Put. Rutin content, however, exhibited a different change pattern than other phenolics; it showed a limited increase in the early stages of storage, then tended to decrease again in the following days. This transient increase can be attributed to the short‐term activation of defense metabolism at the beginning of storage. When phenolic acids were examined, it was determined that the contents of ferulic, protocatechic, p‐coumarinic, caffeic, and o‐coumarinic acids decreased during storage. A particularly significant decrease occurred in caffeic acid, but SA and Put applications were found to limit this loss. The highest level of protection was achieved in most phenolic compounds with combination application (Table 3). These results clearly demonstrate that bioregulator applications play a significant role in phenolic stability. The observed decrease in phenolic compounds during storage is closely related to the ongoing metabolic activities and oxidative processes in the post‐harvest period. Increased respiratory metabolism and accumulation of reactive oxygen species (ROS) in the fruit tissue increase the activity of oxidative enzymes such as polyphenol oxidase, leading to the degradation of phenolic compounds (Mittler 2002; Kiralan and Gundogdu 2021). Furthermore, it has been reported that PPO‐induced oxidative reactions reduce phenolic stability, leading to a decrease in antioxidant capacity (Lee and Kader 2000). The effect of SA applications on the protection of phenolic compounds is largely attributed to its activation of the antioxidant defense system. SA is reported to limit ROS accumulation, increase antioxidant enzyme activities, and support phenolic synthesis by regulating phenylpropanoid metabolism (Haider et al. 2020; Fan et al. 2021; Sang et al. 2022). In addition, the suppressing ethylene production and respiration rate slows down the maturation process, thereby reducing the metabolic consumption of phenolic compounds (Ahmed et al. 2017). The effect of Put is explained by the cellular stability‐enhancing properties of polyamines. Put stabilizes cell membranes, reducing lipid peroxidation, limiting oxidative damage, and thus slowing down the degradation of phenolic compounds (Zhang et al. 2003; Baswal et al. 2020). It is also reported that it can support phenolic synthesis processes by contributing to the regulation of phenolic metabolism via the PAL enzyme. The superior protective effect obtained by the combined application of SA and Put can be explained by the fact that these two compounds act through different but complementary mechanisms. When the strengthening of antioxidant defense, the suppression of oxidative enzyme activity, and the preservation of cellular integrity are considered together, metabolic slowing becomes more pronounced and phenolic stability increases (Zhao et al. 2020). Previous studies have shown that apricot fruit is rich in phenolic compounds and that these compounds are directly related to antioxidant capacity (Bureau et al. 2009; Erdogan‐Orhan and Kartal 2011). However, it is widely reported that respiration and oxidative processes continuing in the post‐harvest period cause a decrease in phenolic compounds (Mittler 2002; Kiralan and Gundogdu 2021). Previous studies have reported that SA applications increase antioxidant capacity by regulating phenolic metabolism and limit quality losses during storage (Fan et al. 2021; Ehtesham Nia et al. 2022). Similarly, Put applications have been reported to contribute to the preservation of phenolic compounds such as chlorogenic acid and rutin in different fruit types (Kibar et al. 2021). It has also been noted that the combined application of bioregulators produces stronger physiological effects and enables more effective regulation of phenolic metabolism compared to individual applications (Zhao et al. 2020). In the present study, the SA + Put combination providing the highest phenolic protection is consistent with these literature findings.

**TABLE 3:** Modification in the phenolic compounds content of apricot fruit during storage due to salicylic acid and putrescine treatments (mg 100 g^−1^).

Storage time		Chlorogenic	Gallic	Syringic	Catechin	Rutin
Harvest		187.36 ± 3.05a	3.57 ± 0.06a	1.53 ± 0.07a	5.74 ± 0.11a	6.80 ± 0.08ab
Day 10		170.77 ± 2.05b	3.13 ± 0.07b	1.00 ± 0.07b	5.07 ± 0.06b	7.41 ± 0.28a
Day 20		142.64 ± 2.02c	2.80 ± 0.05c	0.76 ± 0.05c	4.72 ± 0.08c	7.00 ± 0.18ab
Day 30		130.74 ± 2.99d	2.41 ± 0.03d	0.72 ± 0.03c	4.55 ± 0.12c	6.43 ± 0.07b
Salicylic + putrescine and storage time interaction						
Harvest		187.36 ± 3.05a	3.57 ± 0.06a	1.53 ± 0.07a	5.74 ± 0.11a	6.80 ± 0.08de
Day 10	Control	163.89 ± 1.83c	2.85 ± 0.03e	0.79 ± 0.07def	4.94 ± 0.04cde	8.64 ± 0.07a
	SA 1 mM	174.42 ± 0.99b	3.22 ± 0.03bc	1.10 ± 0.07bc	5.05 ± 0.04c	6.86 ± 0.03d
	Put 1 mM	167.82 ± 2.09c	3.15 ± 0.03c	0.86 ± 0.07de	4.98 ± 0.02cd	7.40 ± 0.06c
	SA + Put	176.96 ± 1.06b	3.31 ± 0.03b	1.24 ± 0.08b	5.32 ± 0.08b	6.75 ± 0.03de
Day 20	Control	136.17 ± 1.86f	2.61 ± 0.02g	0.63 ± 0.09f	4.48 ± 0.07f	7.66 ± 0.05b
	SA 1 mM	144.12 ± 1.31de	2.85 ± 0.04e	0.77 ± 0.07def	4.86 ± 0.04de	6.47 ± 0.05fg
	Put 1 mM	140.58 ± 1.03ef	2.74 ± 0.02f	0.72 ± 0.07def	4.58 ± 0.04f	7.20 ± 0.08c
	SA + Put	149.69 ± 3.09d	2.99 ± 0.01d	0.93 ± 0.08cd	4.98 ± 0.01cd	6.67 ± 0.07def
Day 30	Control	120.71 ± 1.29g	2.28 ± 0.03ı	0.68 ± 0.01ef	4.08 ± 0.07g	6.17 ± 0.10h
	SA 1 mM	136.93 ± 1.10f	2.49 ± 0.02h	0.72 ± 0.07def	4.78 ± 0.02e	6.53 ± 0.05fg
	Put 1 mM	125.86 ± 1.90g	2.40 ± 0.02h	0.66 ± 0.06ef	4.43 ± 0.03f	6.41 ± 0.08g
	SA + Put	139.47 ± 2.26ef	2.46 ± 0.07h	0.81 ± 0.08def	4.90 ± 0.06cde	6.62 ± 0.10ef
ANOVA						
*F* (storage time)		71.79***	49.36***	18.38***	14.72***	4.37*
*F* (SA × Put × storage time)		122.22***	137.02***	13.63***	55.28***	97.77***

Storage time		Ferulic	Protocatechuic	P‐coumaric	Caffeic	o‐coumaric
Harvest		0.91 ± 0.05a	0.77 ± 0.01a	0.94 ± 0.03a	2.10 ± 0.10a	0.83 ± 0.01a
Day 10		0.82 ± 0.01b	0.63 ± 0.01b	0.92 ± 0.02a	1.64 ± 0.03b	0.74 ± 0.01b
Day 20		0.78 ± 0.02c	0.50 ± 0.03c	0.80 ± 0.01b	1.31 ± 0.09c	0.62 ± 0.01c
Day 30		0.68 ± 0.02d	0.39 ± 0.02d	0.69 ± 0.02c	1.01 ± 0.07d	0.56 ± 0.01d
Salicylic + putrescine and Storage time interaction						
Harvest		0.91 ± 0.05a	0.77 ± 0.01a	0.94 ± 0.03a	2.10 ± 0.10a	0.83 ± 0.01a
Day 10	Control	0.79 ± 0.03bcd	0.60 ± 0.01cd	0.85 ± 0.02bc	1.58 ± 0.07bc	0.70 ± 0.01d
	SA 1 mM	0.82 ± 0.03abc	0.62 ± 0.01c	0.95 ± 0.03a	1.64 ± 0.02bc	0.75 ± 0.00c
	Put 1 mM	0.83 ± 0.04ab	0.62 ± 0.02c	0.91 ± 0.01ab	1.60 ± 0.07bc	0.74 ± 0.01c
	SA + Put	0.84 ± 0.00ab	0.68 ± 0.01b	0.95 ± 0.01a	1.73 ± 0.06b	0.77 ± 0.00b
Day 20	Control	0.73 ± 0.03cde	0.38 ± 0.01gh	0.79 ± 0.02cd	0.95 ± 0.06g	0.57 ± 0.00g
	SA 1 mM	0.77 ± 0.03bcd	0.54 ± 0.01e	0.81 ± 0.01cd	1.45 ± 0.08cde	0.65 ± 0.01e
	Put 1 mM	0.79 ± 0.03bcd	0.51 ± 0.02e	0.79 ± 0.02cd	1.33 ± 0.07de	0.60 ± 0.00f
	SA + Put	0.81 ± 0.02bc	0.56 ± 0.02de	0.82 ± 0.04cd	1.51 ± 0.07cd	0.66 ± 0.00e
Day 30	Control	0.63 ± 0.02e	0.32 ± 0.02ı	0.63 ± 0.03f	0.84 ± 0.05g	0.53 ± 0.00ı
	SA 1 mM	0.70 ± 0.03de	0.41 ± 0.00g	0.71 ± 0.02ef	1.05 ± 0.07fg	0.57 ± 0.00g
	Put 1 mM	0.68 ± 0.04e	0.36 ± 0.01hı	0.67 ± 0.03f	0.90 ± 0.06g	0.55 ± 0.00h
	SA + Put	0.71 ± 0.02de	0.46 ± 0.03f	0.76 ± 0.03de	1.25 ± 0.11ef	0.60 ± 0.00f
ANOVA						
*F* (storage time)		22.14***	34.58***	35.57***	27.11***	68.42***
*F* (SA × Put × storage time)		6.27**	80.61***	16.81***	27.5***	359.81***

*Note:* Different letters in the same column indicates statistical differences at *p* ≤ 0.05.

*p ≤ 0.05, **p ≤ 0.01, and ***p ≤ 0.001, respectively.

### 3.7 PCA Analysis

**FIGURE 2:**
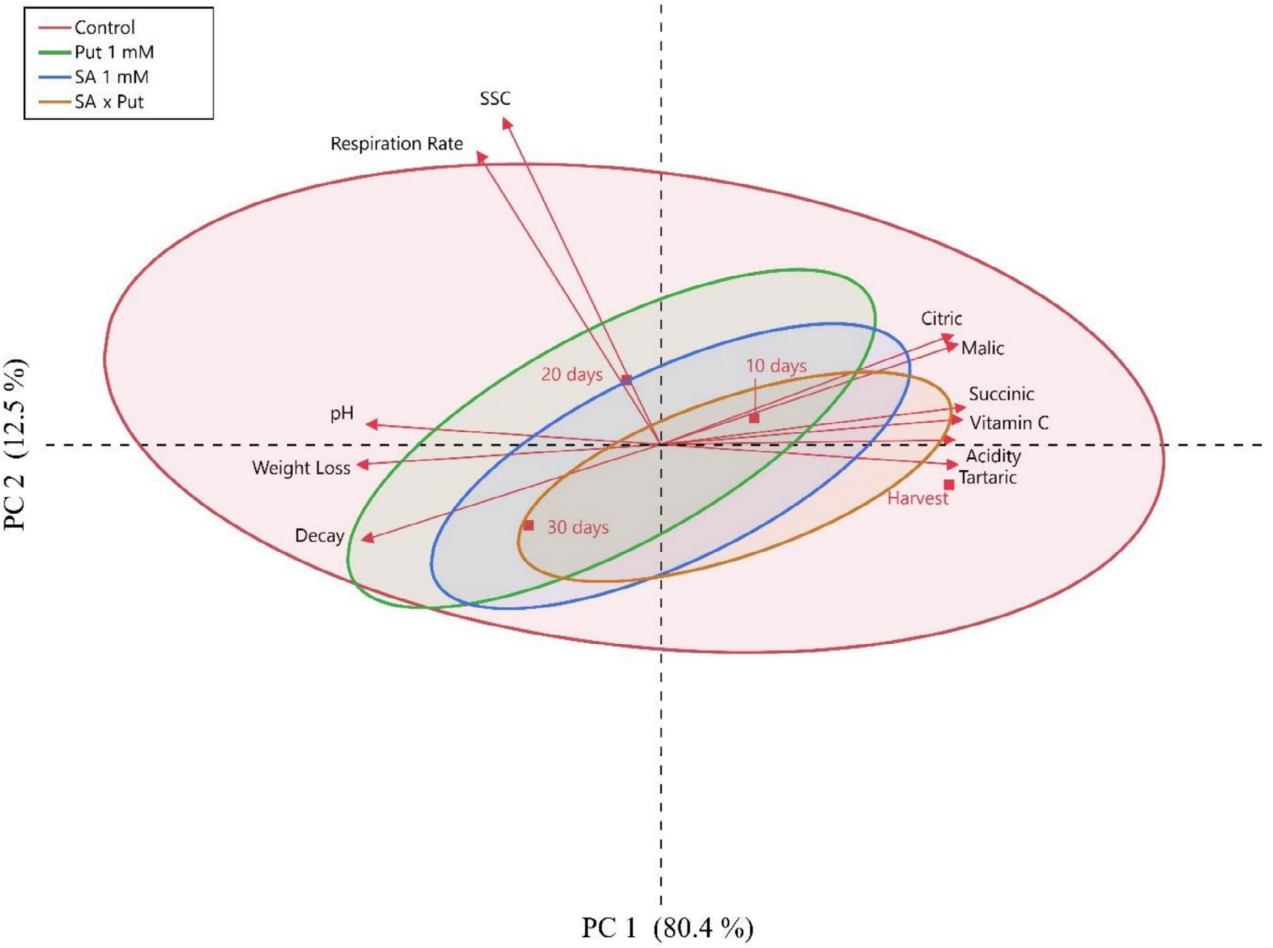
Depending on the processing methods applied, the relationships between changes in quality characteristics and organic acid content of apricot fruits during storage are identified using principal component analysis (PCA).

Principal component analysis (PCA) clearly revealed the holistic effects of storage time and treatments on the quality characteristics and organic acid profile of apricot fruit. The first principal component (PC1) represented storage‐dependent metabolic changes and showed that the samples diverged significantly from harvest to the end of storage. This separation was associated with the decrease in organic acids and changes in quality parameters as storage progressed. The second key component (PC2) reflected the effect of the treatment; while control samples were located closer to variables associated with quality loss, fruits treated with SA (SA) and Put (Put) were found to be associated with parameters that preserved quality. In particular, the clustering of the SA + Put combination in the same region as organic acids and quality indicators indicated that this treatment regulated metabolic processes in a more balanced way. Overall, PCA results indicate that storage time is a key determinant of quality variations, but bioregulator applications particularly combination applications limit post‐harvest quality losses by maintaining biochemical stability (Figure 2).

**FIGURE 3:**
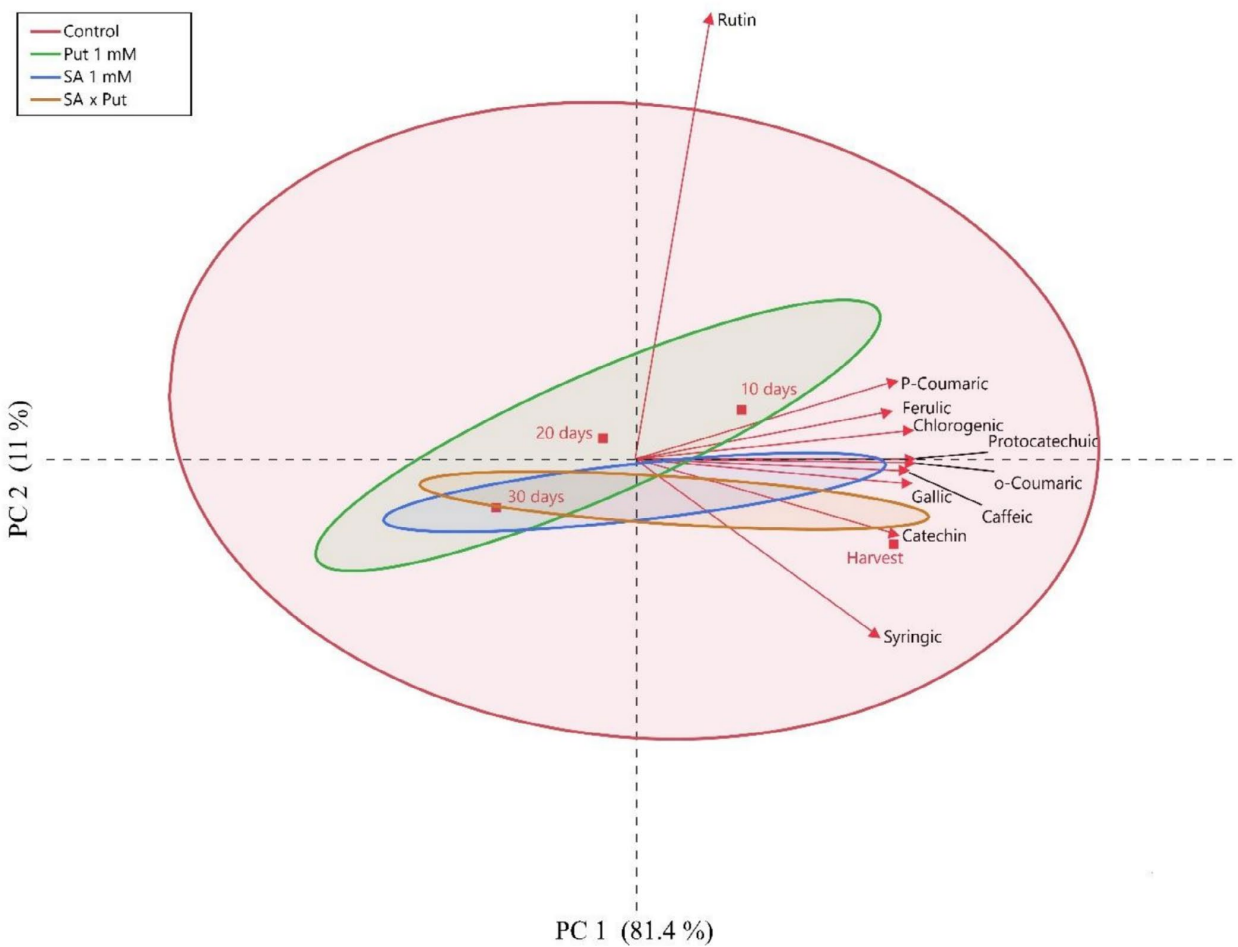
Depending on the processing methods applied, the relationships between changes in phenolic content of apricot fruits during storage are identified using principal component analysis (PCA).

Figure 3 shows the effects of different treatments on the phenolic compound profile of apricot fruits during storage, revealed by principal component analysis (PCA). According to the PCA results, the first principal component (PC1) explains 81.4% of the total variation, whereas the second principal component (PC2) explains 11%. This indicates that the changes in phenolic compounds largely occur along the PC1 axis, and the model represents the relationship between the variables with high accuracy. An examination of the graph reveals that the vast majority of phenolic compounds are positioned in the same direction, indicating a positive correlation between these compounds. The shift in the sample points from the harvest position towards the phenolic compound vectors as storage time progresses demonstrates that the storage process has a significant impact on phenolic metabolism. When evaluated in terms of applications, the wide distribution of the control group indicates higher variation in phenolic content and biochemical dysregulation. In contrast, samples of Put and SA applications formed narrower clusters, revealing the stabilizing effect of these applications on phenolic compounds. Specifically, the alignment of the SA + Putresin combination with phenolic compounds indicates that this application is more effective in preserving antioxidant phenols. Consequently, PCA analysis clearly shows that the applications contribute to the preservation of phenolic compounds in apricot fruit during storage and that the combination application is the most successful method in reducing quality loss.

### 3.8 Classification of Applications in Terms of Fruit Quality Characteristics

**FIGURE 4:**
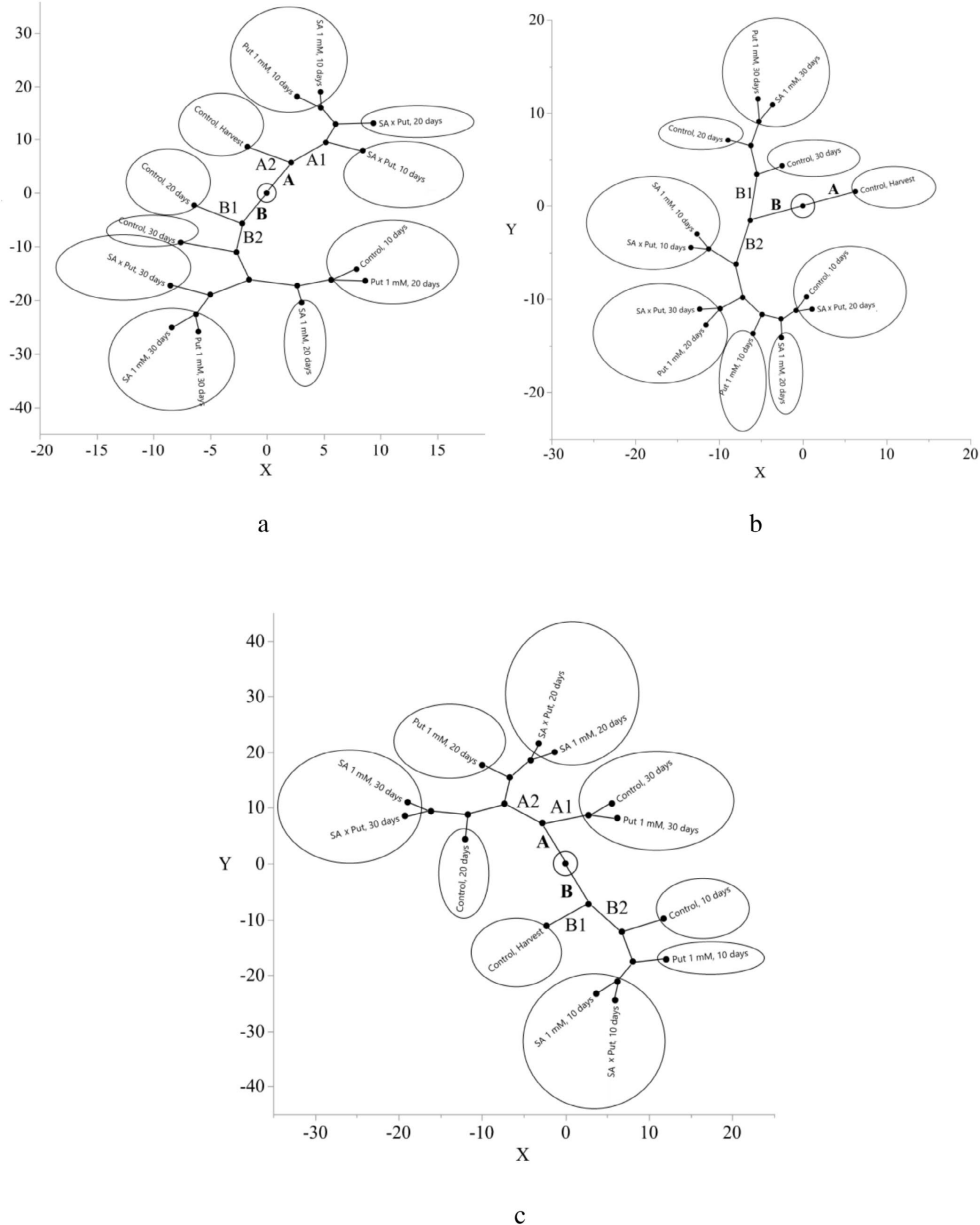
Classification of applications in terms of fruit quality characteristics (a), organic acid content (b), and phenolic compound content (c).

Figure 4 shows the classification of apricot fruit quality characteristics (a), organic acid content (b), and phenolic compound profile (c) during storage using a multivariate analysis approach with different treatments. The graphs allow for a holistic assessment of the overall effect of the treatments on biochemical and quality parameters. Figure 4a shows that the parameters related to fruit quality characteristics differ significantly depending on the treatments. The relationships between firmness, total soluble dry matter, titratable acidity, and color parameters indicate that the treatments directly affect storage performance. The fact that the control samples were positioned further away from the quality parameters revealed that quality loss occurred more rapidly during storage, whereas the formation of more compact clusters in the treated groups indicated that physiological deterioration was delayed. In particular, the alignment of the combination application with the quality indicators showed that it was more effective in preserving fruit texture and overall commercial quality. When the classification of organic acid contents is evaluated in Figure 4b, it has been determined that malic acid, citric acid, and other organic acids exhibit different trends according to the applications. Considering that organic acids are closely related to respiratory metabolism during storage, the more variable distribution in terms of acid profile of the control group suggests that metabolic consumption occurs more rapidly. In contrast, the clustering of the treated samples around specific acid vectors indicates that the applications regulate the respiration rate and limit organic acid loss. Figure 4c clearly shows the classification results of phenolic compounds, revealing the effect of the treatments on antioxidant metabolism. The grouping of most phenolic compounds in the same direction indicates a positive correlation between these compounds, whereas the proximity of the treatment groups to these vectors suggests that phenolic stability is maintained. The relatively distant position of the control group from the phenolic compounds suggests that phenolic degradation is more pronounced due to storage stress. Overall, the classification analyses presented in Figure 4 show that the treatments significantly affect both the quality characteristics and the biochemical components of apricot fruits. In particular, the combination application appears to provide a stronger protective effect compared to other applications in terms of quality parameters, organic acid balance, and the preservation of phenolic compounds. These findings reveal that post‐harvest applications contribute to the sustainability of fruit quality by modulating metabolic processes during storage.

### 3.9 Correlation Analysis

**FIGURE 5:**
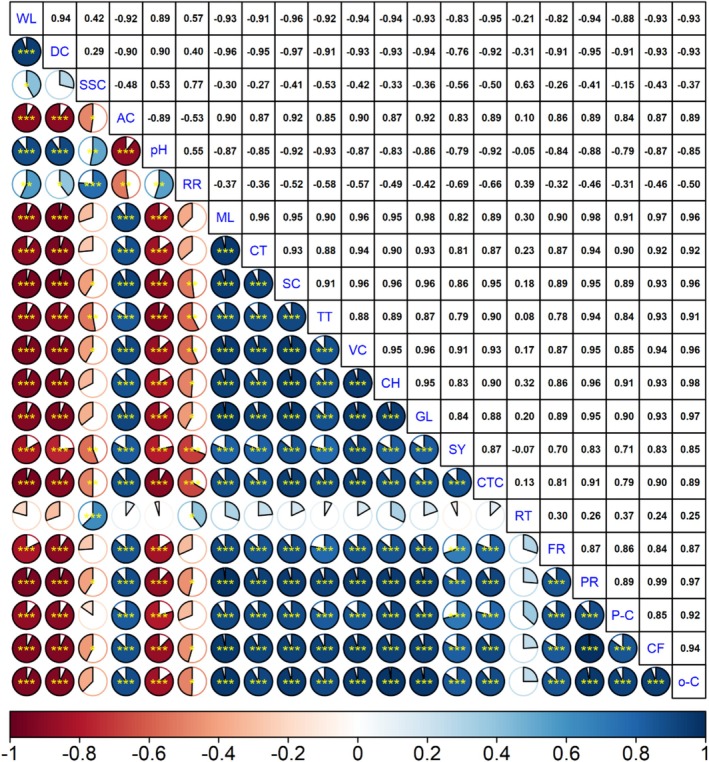
The correlation between fruit quality characteristics, organic acids and phenolic compounds, and depending on the treatment. The color scale that disappears from dark to light shows the correlation values between −1 and +1. *, **, and ***Significance at *p* ≤ 0.05, *p* ≤ 0.01, and *p* ≤ 0.001, respectively. AC, acidity; CF, caffeic acid; CH, chlorogenic acid; CT, citric acid; CTC, catechin; DC, decay; FR, ferulic acid; GL, gallic acid; ML, malic acid; o‐c, o‐coumaric acidP‐C, P‐coumaric acid; PR, protocatechuic acid; RR, respiration rate; RT, rutin; SC, succinic acid; SSC, soluble solid contents; SY, syringic acid; TT, tartaric acid; VC, vitamin C; WL, weight loss.

Figure 5 shows the correlation analysis of the relationships between quality characteristics, organic acids, and phenolic compounds in apricot fruits during storage, depending on different treatments. The correlation matrix was interpreted using coefficients ranging from −1 to +1, with darker shades representing strong positive or negative relationships and lighter shades representing weaker relationships. Furthermore, *, **, and ***symbols indicate statistical significance at *p* ≤ 0.05, *p* ≤ 0.01, and *p* ≤ 0.001, respectively. When examining fruit quality parameters, the positive and significant correlations found between weight loss (WL), decay rate (DC), and respiration rate (RR) suggest that physiological deterioration mechanisms progress in a related manner during the storage process. In contrast, the negative correlation between quality indicators related to hardness and acidity and weight loss and decay parameters reveals that maintaining structural integrity is critical for storage performance. The relationships between SSC and some metabolic parameters show that sugar accumulation and metabolic activity change together as maturation progresses. When evaluated in terms of organic acids, the strong positive correlations found between malic acid (ML), citric acid (CT), and tartaric acid (TT) suggest that these acids are regulated by similar metabolic pathways during storage. The negative correlation of organic acids with respiration rate and weight loss supports the idea that these acids are consumed as substrates due to increased metabolic activity. This explains the changes in taste and acidity balance that occur as storage progresses. When phenolic compounds were examined, the significant positive correlations found between chlorogenic acid (CH), gallic acid (GL), catechin (CTC), rutin (RT), ferulic acid (FR), and caffeic acid (CF) indicate that these antioxidant compounds exhibit a simultaneous metabolic response to stress conditions. The fact that most phenolic compounds show a negative correlation with decomposition rate and respiration rate indicates that high phenolic content plays a role in reducing oxidative stress and delaying tissue degradation. Furthermore, the positive relationships between vitamin C (VC) and phenolic compounds demonstrate that the antioxidant defense system works in an integrated manner. Overall, correlation analysis reveals that quality loss during storage is associated with increased respiratory activity and weight loss, whereas the preservation of organic acids and phenolic compounds plays a critical role in maintaining fruit quality. It appears that the treatments modulate the relationships between these parameters, maintaining metabolic balance and improving storage performance, particularly through the preservation of antioxidant compounds.

## 4 Conclusion

This study revealed that SA and Put applications significantly regulated post‐harvest physiological and biochemical changes in apricot fruit. Quality losses observed during storage were primarily associated with increased respiration activity, oxidative stress, and metabolic depletion. SA and Put applications slowed down these processes, delaying fruit senescence, reducing weight loss and decay development, and contributing to the preservation of SSC, acidity balance, and tissue stability. Storage‐related decreases in organic acids, ascorbic acid, and phenolic compounds were significantly limited by bioregulator applications. Specifically, the SA + Put combination provided the highest biochemical stability thanks to the strengthening of the antioxidant defense system, the preservation of cell membrane stability, and the suppression of ethylene‐related metabolic processes. Multivariate analyses clearly demonstrated that the combination application was strongly correlated with quality parameters and antioxidant metabolism. Consequently, the combined application of SA and Put stands out as an effective and feasible post‐harvest strategy for preserving phenolic metabolism, reducing oxidative damage, and extending shelf life in apricot fruit. This approach offers a sustainable method that can contribute to both reducing quality losses in commercial storage processes and preserving the nutritional value of the fruit.

## Author Contributions

**Erdal Aglar:** validation, resources, software, writing – original draft, writing – review and editing. **Mustafa Sakaldaş:** conceptualization, methodology, software, formal analysis, investigation, writing – original draft, visualization, data curation. **Fatih Şen:** conceptualization, methodology, software, data curation, investigation, formal analysis, visualization, writing – original draft. **Muttalip Gundogdu:** validation, resources, software, writing – original draft.

## Conflicts of Interest

The authors declare no conflicts of interest.

## Data Availability

The data that support the findings of this study are available from the corresponding author upon reasonable request.
